# Creating tissue on chip constructs in microtitre plates for drug discovery†

**DOI:** 10.1039/c8ra00849c

**Published:** 2018-03-06

**Authors:** N. P. Macdonald, A. Menachery, J. Reboud, J. M. Cooper

**Affiliations:** ARC Centre of Excellence for Electromaterials Science (ACES), Australian Centre for Research on Separation Science (ACROSS), School of Chemistry, Faculty of Science, Engineering and Technology, University of Tasmania Private Bag 75 Hobart TAS 7001 Australia; The Advanced Microfluidics and Microdevices Laboratory (AMMLab), New York University Abu Dhabi P.O. Box 129188 Abu Dhabi UAE; Division of Biomedical Engineering, School of Engineering, University of Glasgow Rankine Building, Oakfield Avenue Glasgow G12 8LT UK jon.cooper@glasgow.ac.uk

## Abstract

We report upon a novel coplanar dielectrophoresis (DEP) based cell patterning system for generating transferrable hepatic cell constructs, resembling a liver-lobule, in culture. The use of paper reinforced gel substrates provided sufficient strength to enable these constructs to be transfered into 96-well plates for long term functional studies, including in the future, drug development studies. Experimental results showed that hepatic cells formed DEP field-induced structures corresponding to an array of lobule-mimetic patterns. Hepatic viability was observed over a period of 3 days by the use of a fluorescent cell staining technique, whilst the liver specific functionality of albumin secretion showed a significant enhancement due to the layer patterning of cell lines (HepG2/C3A), compared to 2D patterned cells and un-patterned control. This “build and transfer” concept could, in future, also be adapted for the layer-by-layer construction of organs-on-chip in microtitre formats.

## Abstract 2

We report upon a novel coplanar dielectrophoresis (DEP) based cell patterning system for generating transferrable hepatic cell constructs, resembling a liver-lobule, in culture.

## Graphical abstract

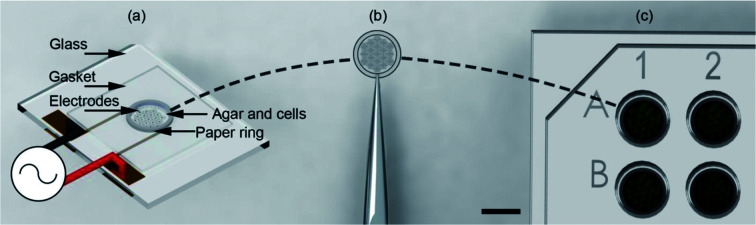

## Introduction

Cellular patterning is an enabling technology and can be used to build cell-based architectures from the bottom up. The technique has been widely used in a variety of applications associated with organ replacement,^1^ medical diagnostics^2^ and drug delivery.^3^ Such studies have demonstrated that the replication of both *in vivo* geometries and dimensional scale contributes to the recapitulation of *in vivo*-like cell phenotypic architectures within *in vitro* constructs.^4^ Engineered microstructures and patterns based upon a specific organ have also been shown to strongly enhance the functionality of co-cultures of different cell-types, aimed at recapitulating the activity of the organ.^4–6^

Organs-on-chip or tissue-on-chip are microengineered platforms that integrate physiologically relevant local environments within cultured biological tissues.^7–9^ A growing number of different anatomical structures have been established, including the spleen,^10^ lungs,^11^ interconnected neurons^12^ and intestinal villi.^13^ Their use is supported by a wide range of modelling and control systems such as whole-body pharmacokinetic pharmacodynamics (PK–PD) modelling^14^ to study drug activities for example. Engineered micro-organs have also shown promise to both improve the accuracy and reduce development costs of drug compounds.^15^ The liver is an essential organ when considering toxicity studies in the process of drug development and the reconstruction of functional tissue assemblies has the potential to provide highly relevant surrogate models.^16,17^

Patterning biomimetic liver-on-a-chip has been achieved using a variety of microengineering and microfabrication technologies.^18–23^ Successfully generating the building block of the liver (lobules – consisting of a unit of hepatocytes lined by endothelial cells – hepatic cords) has been an important development.^24,25^ A number of devices have used trapping systems to reform various cell types into hepatic cords, as demonstrated by using microfluidic channels lined with micro pillars fabricated to culture hepatocytes into hepatic cords.^21^ Such micro-engineered devices have also been used to test drug toxicity *in vitro*,^22^ and co-culture A549 and C3A cells.^26^ Endothelial-like microfluidic barriers can also be fabricated to align into a hepatocyte arrangement.^18,20^ The complexity of these devices has resulted in research into microfluidic on-chip construction of lobule like structures.^19^ Hepatocytes and nonparenchymal cells have also been accurately arranged into hepatic sinusoids by fabricating cell-laden hydrogel microfibers.^23^

Dielectrophoresis (DEP)^27,28^ has also previously been used to successfully engineer liver lobule patterns on-chip with both human liver cell line (HepG2)^29^ and human umbilical vein endothelial cells (HUVECs).^30^ The 3D patterning of rat hepatocytes in various biomimetic microstructures within a photopolymerisable polyethylene glycol (PEG) hydrogel has also been demonstrated.^31^ These successful DEP patterning systems however are limited by their reliance upon specific device designs, which can only be used once (one culture, one device). Further, they do not provide a re-useable platform for patterning cells within a 3D hydrogel, and perhaps most importantly, the resulting geometries are not appropriate for high throughput methods, commonly used within drug screening laboratories.

Within cell-tissue patterns, full biological activity only appears in a coherent and functional fashion after being cultured for several days. Many microdevice formats currently proposed often do not allow such longer-term incubation, for example due to the fragility of the hydrogel layer or as a consequence of the need for an iterative disassembly/re-assembly.^31–33^ To date this has prevented a seamless transfer of the tissue structures to higher throughput conventional culture dishes, thereby restricting the use of DEP-patterned hydrogels in applications where a high number of experiments are required such as in drug development applications.

**Fig. 1:**
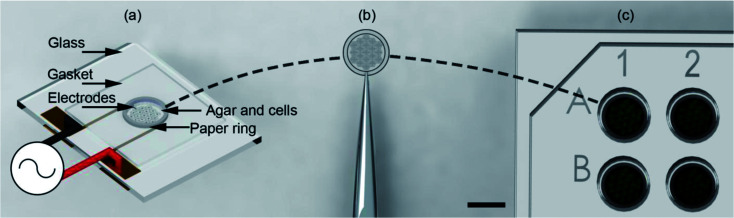
Illustration of the “build and transfer” system. (a) Utilising a single plane biomimetic DEP electrode arrangement, C3A human liver cells where patterned within an agar hydro-gel supported by a 6.2 mm OD, 5 mm ID paper ring. The system was heated during patterning to maintain the liquid state of the agar, then cooled to cure the agar into a gel. Patterning was induced by positive DEP through a 10 MHz, 10 Vpp AC signal. (b) Supported by a paper-ring substrate, the patterned cells were removed by tweezers. (c) Patterned cells were then immediately placed in a 96-well plate for incubation and subsequent biological analysis. Scale bar is 5 mm.

Here we show that the use of support structures, conveniently manufactured in paper matrices, enables the assembly of the cell-tissue constructs within a microdevice, (using DEP in an open-top co-planar electrode configuration) which can then be followed readily by its extraction and its positioning into a 96 well plate for subsequent culture and analysis (Fig. 1).

Previously, hydrogels and agar have both been shown to be useful for tissue engineering, providing a suitable microenvironment for mammalian cells.^33^ For example, agar scaffolds have been developed which enable HepG2 liver cell culture^34^ providing both biocompatibility and porosity and allowing the ready diffusion of nutrients and gases to cells at temperatures above 30 °C. Recently, thin hydrogel films have been used to provide an alternative to conventional extracellular matrix (ECM) for culture with a range of cell types under perfusion.^35^

In this paper, we now build upon this previous work, and use DEP patterning in hydrogels for the creation of a liver-like biomimetic architecture. The technique has a number of advantages in that the devices are scalable into higher throughput formats and have a co-planer design such that they are 96-well plate compatible. The system also maintains a cell pattern in the hydrogel, with the potential to stack multiple layers with different patterns, cell types and hydrogels. To create complex 3D architectures in future, the cell-laden gel layers could be partially melted to increase cell–cell contacts. The equipment also has the advantage that it is reusable.

We now show that liver specific functions were enhanced in this new configuration (illustrated here with the secretion of albumin). The system uses an innovative method, integrating temperature control with Peltier coolers, laser cut paper rings and patterning of cell within a hydrogel, to produce a reusable cell patterning system (“build”) that can enable the assembly of layers of biomimetic cell-laden hydrogels either with microfluidic or static culture conditions.

Further, we demonstrate that cell viability was maintained over a period of three days, when cells were patterned within the hydrogel. The C3A liver cells exhibited a more liver-like functional behaviour, as illustrated by their increased secretion of albumin, with respect to non-patterned cells.

## Materials and methods

### Materials

Cell lines HepG2/C3A were acquired from ATCC, UK. All chemicals were supplied by Sigma-Aldrich, UK unless otherwise stated. Eagle's Minimum Essential Medium (EMEM) was supplied by ATCC, UK and EMEM was supplemented with 10% fetal bovine serum. Trypsin 0.25%, dimethyl sulfoxide (DMSO) and phosphate buffered saline (PBS) were of a grade compatible with cell culture. DEP buffer comprised 9% sucrose, 0.3% dextrose, 3 mM HEPES in DI water; conductivity 13 mS m^−1^ pH 7.4, osmolality 300 mOsm. Albumin Human ELISA kit (ab108788) was obtained from Abcam, Cambridge, UK. Trypan Blue solution, 0.25% and LIVE/DEAD® Viability/Cytotoxicity Kit, for mammalian cells were obtained from Life Technologies, UK.

Low gelling temperature agarose (Type IX-A: Ultra-low Gelling Temperature) for cell culture was obtained from Sigma-Aldrich. Petri dishes, 6, 24 and 96-well plates, sterile single use pipettes, Eppendorf tubes, and microscope slides were obtained through Fisher Scientific, UK. Whatman no. 114 (USA) filter paper was obtained through Sigma-Aldrich. Nescofilm was obtained from Alfresa Pharmaceutical Corp, Osaka, Japan. To perform DEP, a 50 MHz TG5011 LX1 frequency generator (Thurlby Thandar Instruments Ltd, UK) was used. Temperature control was maintained using a pair of Peltier coolers (MULTICOMP – MCPE-071-10-13, 19.1 W, 1639748) supplied by Farnell, controlled by a dual-rail current supply EX 354D 280 W (Thurlby Thandar Instruments Ltd, UK).

A Hewlett–Packard (HP) DesignJet was used to fabricate the cover for the system. Parts were left in a water bath at 60 °C with a mixture of sodium percarbonate, carbonate and bicarbonate with de-ionised (DI) water. Passive cooling solutions were provided by a selection of heatsinks: heatsink 6.8 °C W^−1^ (36-0292) from Rapid Electronics, UK and Zalman CNPS7000a-CU from Zalman, USA. Thermal connection was made by a proprietary paste (36-0400) from Rapid Electronics, UK. Standard mechanical grade silicon thickness 500 μm, 4 inch wafers were supplied by University Wafer, USA. Additionally, gold (Au) and titanium (Ti) wire for resistive heating evaporation deposition were supplied by the James Watt Nanofabrication Centre and were obtained from Goodfellows UK.

### Cell culture and analysis

Cell lines were cultured in a HERA cell 150 incubator (Thermo Scientific) and maintained at 37 °C with 5% CO_2_. Antibiotics were used prophylactically in cell culture maintenance however, it has been observed that they cause disruption of cellular functions including cell morphology, cellular degeneration and cell death.^36^ For example, hepatocytes have been observed to have reduced metabolic function when cultured with penicillin, a commonly used antibiotic in cell culture.^37^ As toxicity studies using hepatic cell lines are reliant on reproducible data from protein synthesis, antibiotics were not used in this work. The live/dead kit viability kit was used according to the supplier instructions to assess the health of the C3A cells by simultaneously staining the living and dead cells.

### Albumin secretion analysis

The cell culture medium was collected from the wells and stored in 1.5 ml Eppendorf tubes at 4 °C. The albumin concentration was measured using the human albumin assay kit following the supplied protocol (ab108788, Abcam, Cambridge, UK). Assays were performed in duplicate for each sample. Medium samples including 2D control, agar control, and pattern samples were collected each day and stored at −20 °C until assayed. The data from the plate-reader was averaged across 4 separate experiments. Analysis of variance (ANOVA) was performed using GraphPad Prism v7.03 (GraphPad Software, Inc., U.S.A.).

### Characterisation

Measurements for albumin production were taken with a BioTek ELx808 high throughput plate reader at 450 nm and 520 nm accordingly. Data was acquired using the supplied software package Gen5™. Fluorescence microscopy was carried out on a Zeiss D.1 Axio Observer. A mercury lamp (HBO 100) was used to excite fluorophores, and filtered using fluorescein isothiocyanate (FITC) (XF100-2) and rhodamine (Rho) (XF101-2) filters; excitation at 475 and 525 nm, emission at 535 and 565 respectively. The microscope objectives used were: Zeiss EC Plan-NEOFLUAR ×5, ×10 and ×20. Images were captured using an Andor tech iXOn EM+ camera with a 60N-L 1.0× collar. Images were collected and processed using the ImageJ software package.^38^

Osmolarity of solutions was measured using an Advanced Micro Osmometer 3300, calibrated using standards of 850, 290 and 50 mOsm kg^−1^ supplied by Advanced Instruments, UK. Conductivity was measured using a Jenway 4071 conductivity meter (288-8199) obtained from RS, UK. pH measurements were taken using a Hanna Instruments pH meter and calibrated using standards of 4.0, 7.0 and 10.0 pH (Fisher). Thermal imaging was performed using a Ti 25 Fluke thermal camera from Fluke, Norwich, UK. Direct thermal readings were taken from devices using a thermocouple CM1200T (IEC 584-3)T from Caltek Industrial (H.K.) Ltd, Hong Kong.

### Electrode fabrication

DEP electrodes, in patterns that mimicked the liver lobule, were fabricated using photolithography and metallisation to define electrodes of Pt (10 nm)/Au (100 nm) suitable for DEP, connectors were then soldered to the electrodes. The fabrication process used for the electrodes in this work follow the well-defined photolithographic techniques.^39^

### 3D design

2D and 3D CAD drawing was completed in SolidWorks 2011 ×64 (Dassault System SolidWorks Corp, Concord, MA, USA), completed .stl files were verified using Solid Edge (Siemens PLM Software, UK). Completed .stl files were then processed by HP Designjet 3D Software Solution for the HP DesignJet, ProJet®.

### Hydrogel and paper substrate preparation

C3A cells were harvested from 70% confluent cultures and detached from the surface of 25 ml culture flasks using 0.25% trypsin–EDTA. The cell solutions were diluted with 5 ml of warmed (37 °C) EMEM and then pipetted into a 15 ml centrifugation tube. Cells were centrifuged for 3 minutes at 1500 rpm (312 × *g*) to form a soft cell pellet. Waste media was gently removed from the tube using pipetting, the final solution being removed using a 200 μl pipette in order to reduce transfer of extra ions into the final solution. The pellet was then pipetted with 5 ml of DEP buffer. A low conductivity was selected to maximise the dielectrophoretic forces exerted on the cells.^40^ The suspended cells were then centrifuged again at the same settings; the process was repeated 3 times. Finally, 200 μl of DEP buffer was added to the cell pellet to yield a cell concentration of 2.5 × 10^6^ cell/200 μl. This concentration was selected to provide 125 × 10^5^ cells/10 μl, which was the designed volume used to fabricate the micro liver.

Low-gelling agar was dissolved by boiling with DEP buffer to give a final agar concentration of 2% (w/v). Batches of this buffer were sterilised by autoclave to ensure sterile conditions for the cells. This agar solution was maintained at 37 °C in a water bath until required. A pre-warmed (37 °C) Eppendorf 1.5 ml tube was used to mix 1 : 1, agar 2% solution with 2.5 × 10^6^ cell per ml concentrate DEP buffer; yielding a 1% agar solution of 1.25 × 10^6^ cell per ml with DEP buffer (a concentration corresponding to *ca.* two liver lobules).^41^ This final solution was kept at 37 °C before use in DEP-based cell-patterning experiments.

### Agar and paper substrate manipulation

As shown in Fig. 1(a) the top of the electrodes had a 100 μm gasket made from Nescofilm with a 5 mm diameter hole cut in the centre. This defined the volume of the agar cell solution to be patterned. Around the circumference of this gasket a 5 mm ID, 6.2 mm OD paper ring previously soaked in 2% (w/v) agar was placed. This defined a 7.7 pl volume for electrode patterning. The paper construct was incubated to stop the wicking of 1% agar DEP buffer cell solution into the cellulose matrix. A 10 μl agar cell solution was pipetted into this volume for patterning. Finally, patterned gels were removed from the container with the support of the paper ring using tweezers, Fig. 1(b) and placed inside a 96-well as shown in Fig. 1(c) with growth medium for subsequent cell culture.

### System design and operation

**Fig. 2:**
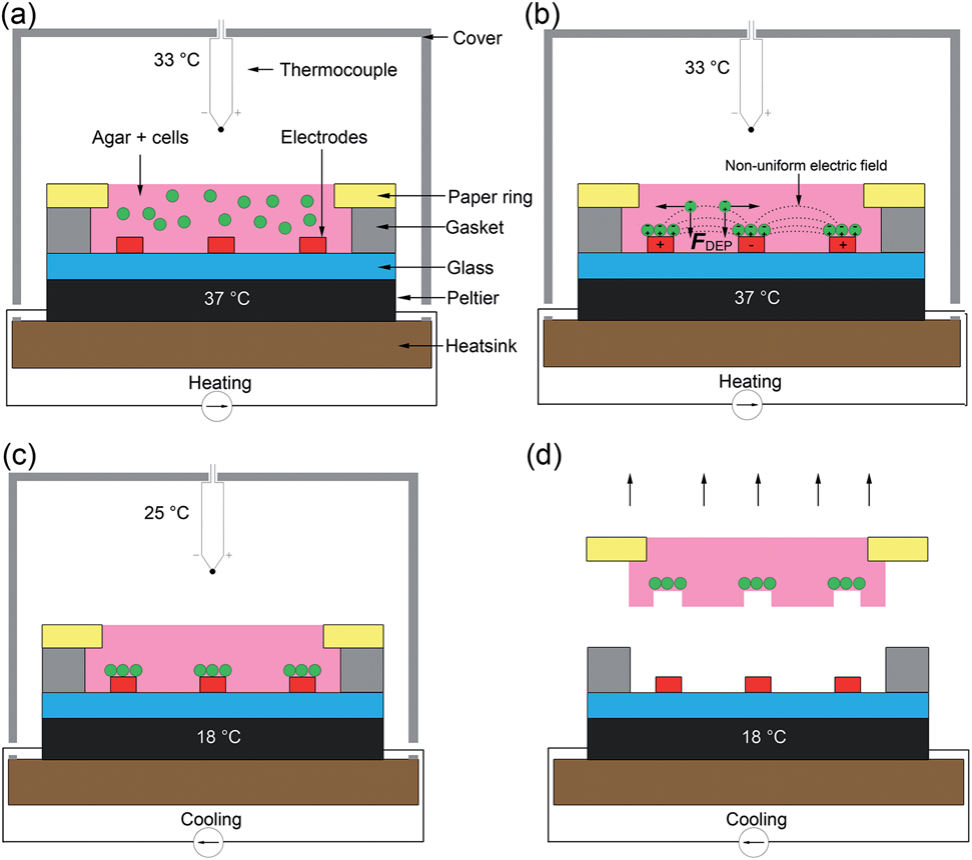
The “build and transfer” system setup and operation for patterning liver like structures within an agar gel with a paper substrate. (a) Warmed 1% agar solution with randomly distributed cells at 37 °C was pipetted into volume designated by the gasket. The Peltier warmed the glass substrate on which the electrodes are situated and enclosed with a cover. (b) Hepatic cells were captured by positive DEP forces (F_DEP) induced by a non-uniform electric field (10 MHz, 10 Vpp). (c) Once patterning was complete, the Peltier was switched to cooling mode dropping the temperature of the agar to 18 °C, curing the gel. Once the air temperature reached 25 °C the AC field was switched off. Agar 1% solution enters gel phase at this temperature. (d) The cells patterned by the DEP forces are held in place by the cured agar. The paper substrate containing a biomimetic micro liver is used to manipulate the patterned cells into a culture system.

The design and operation is illustrated in Fig. 2. Microfabricated Pt/Au electrodes on the surface of a glass substrate were used to build a micro liver in agar with a paper substrate. The substrate was placed on a Peltier cooler. A 3D printed cover, designed in SolidWorks and fabricated with the fused deposition modelling HP DesignJet, was employed to reduce evaporation during the patterning process. A copper heat sink was the base of the system, which was connected to the Peltier reversibly with thermal paste. A drop of deionised (DI) water acted as a thermal interface between the Peltier and the glass. In order to keep the 1% agar-cell solution in a liquid state (viscosity typically 60 mPa s^−1^)^42^ to allow for cell movement into the desired patterns, the temperature was maintained at 37 °C.

Following patterning, the system is then cooled to 18 °C by reversing the current of the Peltier and the AC field is switched off once the air temperature has dropped to 25 °C, at which point the agar gels,^43^ ‘freezing’ the pattern., which was then manipulated through the paper ring and transferred to conventional cell culture plates for long term studies.

## Results and discussion

### Electrode design

**Fig. 3:**
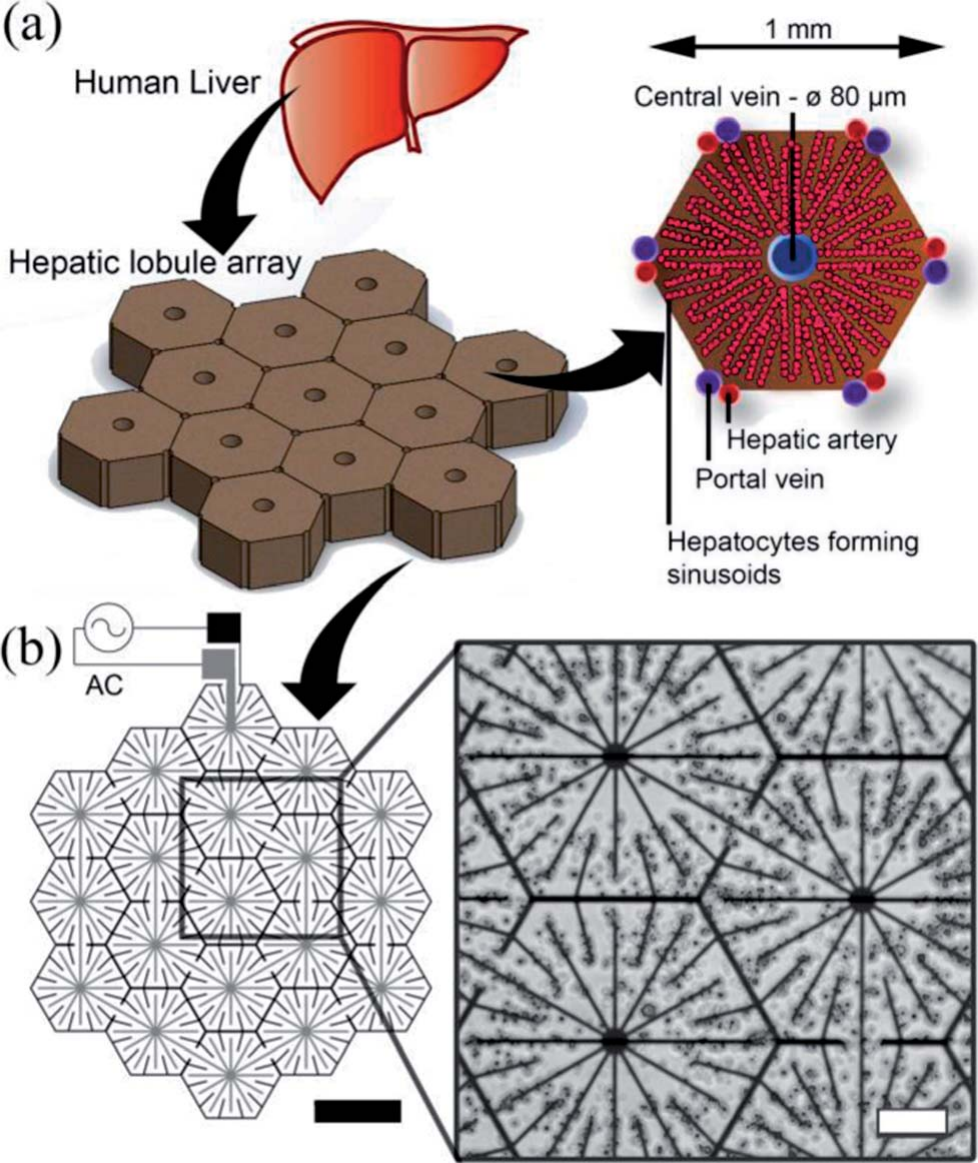
Electrode design. (a) The liver is the largest organ in the body and receives blood from the hepatic portal vein and the hepatic artery. The liver is composed of small units, which can be described in a number of ways. One of the common descriptions is the “classic lobule”.^47^ The lobules are approximately hexagonal in cross-section, with the central vein (80 μm diameter) in the centre surrounded radially by sinusoids constructed of hepatocytes. Microelectrode design for inducing DEP forces to pattern cells into a micro liver lobule array. (a) Using a biomimetic electrode design, hepatocytes are patterned into an array 19 lobules in size using DEP forces. The largest gap is 60 μm between the electrodes. The width of the electrodes is 10 μm, thickness 100 nm. (b) Microscope image showing C3A liver cells being held in place by DEP forces to form liver lobule like structures (10 MHz, 10 Vpp for 2 min). Scale bar is 200 μm.

The human liver consists of two lobes, which are irregular in shape and divided by the falciform ligament Fig. 3(a). In mammals, the hierarchical structure of these lobes finally end in 10^5^ to 10^6^ functional units called lobules.^44^ In cross-section, the hepatocytes are seen to be arranged in plates of one to two cells thick, organised radially around the central vein in cords, as illustrated in Fig. 3(a), separated by sinusoids. Our DEP system design is inspired from this anatomical data, as illustrated in Fig. 3(b): the inner branches of the electrodes (grey) were connected to an alternating current (AC) supply, the outer connection (black) to the ground line. The design uses 19 lobules, giving the array dimensions of 5 × 4.6 mm. This array size was chosen to allow it to fit easily within industry standard 96-well plates (6.86/6.35 mm (top/bottom) in diameter). The 10 μm electrode width replicates the sinusoids in the liver.^45^ The central point of each lobule was designed to be 80 μm in diameter corresponding to the diameter of the centrilobular vein, and similarly for the width (1 mm) and sides (500 μm) of the lobule, also with relation to anatomical data.^46^

Before cell patterning experiments for C3A cells (10–15 μm in diameter), we established the most sensitive signal frequency to use for DEP manipulation. Cells mixed with DEP buffer and agar to make a 1% (w/v) solution were pipetted onto the electrodes. We observed electric-field induced negative DEP, where cells are repelled away from the electrodes, at frequencies close to 10 kHz, while strong positive DEP, where the cells were attracted to the areas of high field gradients, towards the electrodes, was generated at fields over 1 MHz. A voltage amplitude of 10 Vpp was chosen to increase positive DEP forces without any loss of cell viability. These observations are in agreement with previous studies of DEP based cell patterning.^31,48^

The patterned cells, inside the agar, structured on the paper ring, were then cooled below the gelling temperature (25 °C) using the Peltier device and manually transferred to culture plates Fig. 1(a). It is important to note that the cells were mixed with the agar solution, effectively encapsulated once it cured. The patterning process only lasted a few minutes preventing cell sedimentation and attachment. When the paper substrate was manipulated and transferred, all cells were transferred as well together with the gel. Experiments using lower concentrations of agar (0.8, 0.5 and 0.25%) resulted in the gel not attaching to the paper ring.

### Cell viability assessment for DEP cell patterning in agar

**Fig. 4:**
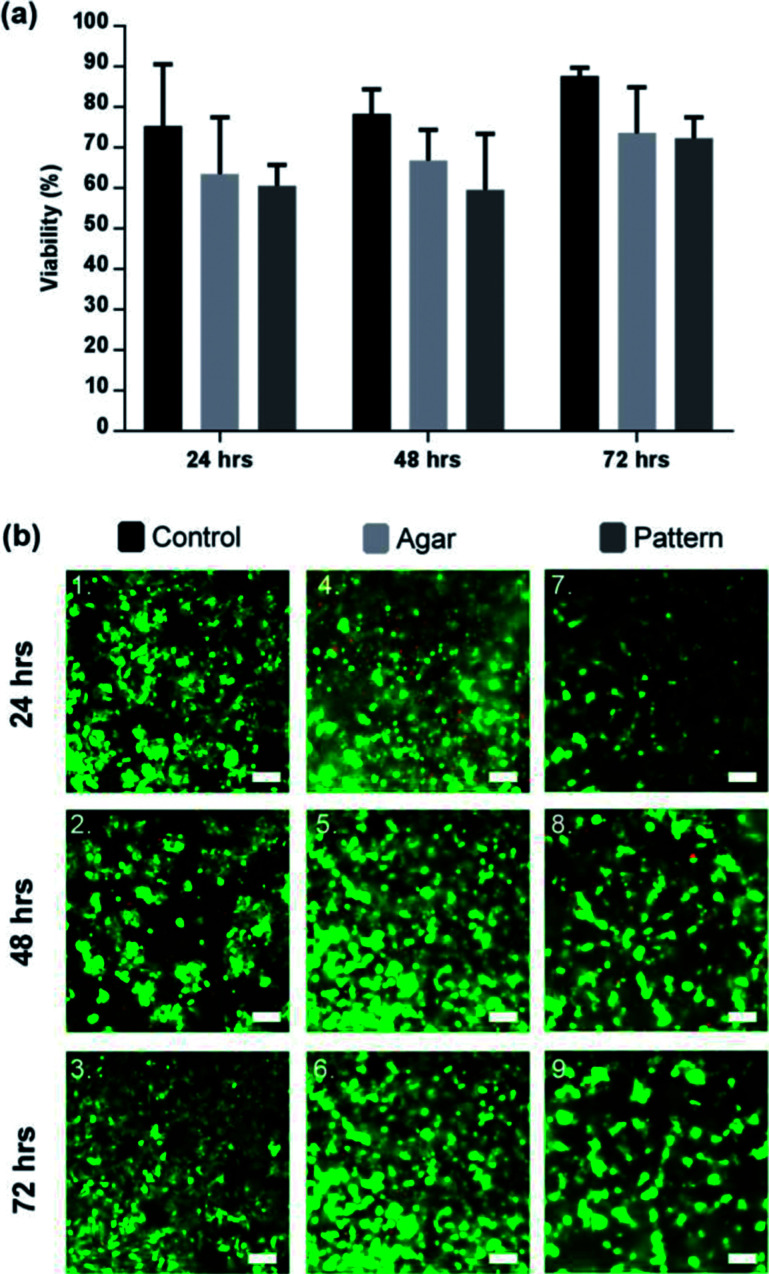
Viability of patterned lobule like structures. (a) Viability of 2D control (black), agar control (light grey), and C3A lobule like structures (dark grey) for a period of 72 h. Cell viability in the control was consistently higher than the agar control or pattern by 72 h (87.64 ± 2.0%). Comparatively, the agar control was observed to be 73.6 ± 11.3% and the pattern was 71.3 ± 5.6%. Data represent the mean ± STD for four independent repeats. Statistically there was no significant differences between the 3 sample groups. *p* > 0.05 (ANOVA). (b) Microscope images of C3A cells after 72 hours of culture. (1–3) 2D sample spread across the surface of the well-plate. (4–6) C3A cells encapsulated within agar gel; cells in close proximity formed small aggregates or spheroids. (7–9) Patterned cells maintained position while forming cords of cells in the shape of the original design. Scale bars 100 μm.

The *in vitro* cell viability was assessed using the mammalian live/dead assay kit, a fluorescence-straining method which stains in green the cytoplasm of live cells and in red the nucleus of dead cells. Fig. 4 shows that the viability of C3A lobule-like structures in agar increased over 72 h to 71.3 ± 5.6% (% green fluorescent cells). Cells cultured in standard and 3D conditions in well plates (cells seeded in agar on the DEP device but without 2D patterning) had a viability of 87.64 ± 2.0%. Images in Fig. 4(b) are a composite of both live cells (green) and dead cells (red) images. Viability data indicates that the DEP patterning system had no significant effect on the cells. Cell density was observed to double after 48 h in 2D controls, faster than for the cultures in gels (both patterned and unpatterned), and in close agreement with previous studies of 3D cell cultures.^31^

Successful patterning with DEP, as observed in Fig. 4(b), led to the development of cell morphologies that are relevant of their functionalities, correlating to the original lobule pattern and fusing with neighbouring cells. They produced dense, cord-like chains of hepatocytes as shown in Fig. 4(b).

Aggregation of cells was observed in the 3D control, however, due to the random distribution of cells through the agar, only spheroid-like aggregates were observed shown in Fig. 4(b), which is in agreement with the literature.^49,50^ Agglomeration of HepG2 has been confirmed to be beneficial for cell function and viability.^51^ While the factors that modulate the hepatocyte phenotype *in vitro* remain to be elucidated, it is accepted that heterotypic cell interaction between hepatocytes modulate cell growth, migration and/or differentiation.^52^

Contrary to previously published 2D DEP strategies in microfluidic systems,^32,53^ our patterns can be maintained for longer periods of time, providing a useful platform for a large range of applications in drug development for example. As shown in our 2D control results, 2D culture puts significant constraints on cell density and the duration of incubation, as cells grow quickly, overcoming the patterns created within less than 48 h.

### Micro liver function assessment *via* albumin secretion

**Fig. 5:**
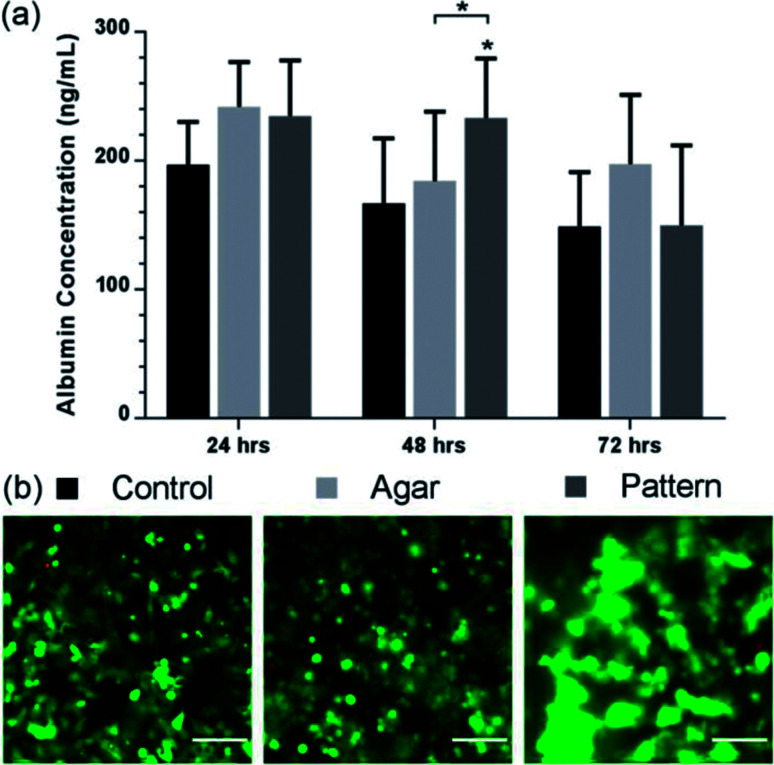
Albumin production of lobule like structures. (a) Albumin value (ng mL^−1^) of control (black), agar control (light grey), and patterned C3A cells (dark grey) over 72 h. Patterned C3A cells had increased albumin secretion after 48 h of culture compared to the control and monolayer control. The control samples show a stable production over the first two days before decreasing. Data represent the mean ± STD for four independent repeats. The (*) indicates statistically significant increase in albumin protein of patterned cells relative to control, and agar 48 h, *p* ≤ 0.05 (ANOVA). (b) Viability images of control, agar control, and patterned cells. Images are a composite of both live cells (FITC-green) and dead cells (rhodamine – red) images. Scale bars 100 μm.

To explore the potential of our method to perform bioassays, we assessed albumin secretion over 3 days, using an ELISA shown in Fig. 5. Albumin secretion is a liver-specific function for hepatocytes, requiring liver-specific gene expression and intact translational and secretory pathways in the cells.^54^ Patterned C3A cells showed statistically-significant increased albumin secretion after 48 h of culture (233 ± 46 ng ml^−1^) compared to the agar control (184 ± 54 ng mL^−1^) and monolayer control (167 ± 51 ng mL^−1^). The control samples showed a stable production over the first two days before decreasing after 72 h. However, the concentration decreased for all conditions after 72 h, while viability of the cultures remained high as observed in Fig. 4(a); this would suggest that the production of albumin had saturated for all three samples rather than cell death causing a reduction.

The albumin concentration observed in the 2D culture after 24 hours was higher than the patterned and control samples, probably due to a higher cell concentration linked to rapid initial proliferation, but patterned cells subsequently have a higher level. These results support the fact that aggregation of hepatocytes is critical to maintaining hepatic cellular functions,^49^ as it has been observed that randomly dispersed low density seeding of cells in agar reduces functionality and viability compared with high density. Tightly packed aggregates with an average diameter of 100 μm have shown increased albumin production compared with randomly dispersed cells.^49^

## Conclusions

Cell patterning techniques to engineer biomimetic cell geometries and to preserve cell-to-cell interactions are an important aspect of constructing artificial organs, for example for toxicity testing. In this work, a novel technique is developed using DEP to pattern cells into the livers' lobule-like structure in agar gels with a paper substrate to facilitate long term studies of both viability, and functionality. The functionality of the liver-lobule structures in agar were investigated by examining the production of albumin, which was increased in the micro liver engineered tissue. The design allows the patterned cells to be removed from the electrode substrate, to be placed within custom culture platforms, for example containing microfluidic channels. Additionally, multiple layers of paper ring micro livers could be constructed to increase the volume and complexity of these tissue-engineered organs into an organ additive manufacturing system.

## Conflicts of interest

There are no conflicts to declare.

## Supplementary Material

RA-008-C8RA00849C-s001
