# Supplementary material for: Creating tissue on chip constructs in microtitre plates for drug discovery
Source: RSC Adv. 2018 Mar 6;8(18):9603–10. doi: 10.1039/c8ra00849c (PMC9078682; doi:10.1039/c8ra00849c)
Supplement: RA-008-C8RA00849C-s001 [file RA-008-C8RA00849C-s001.pdf]

Electronic Supplementary Material (ESI) for RSC Advances.
This journal is © The Royal Society of Chemistry 2018

Creating Tissue on Chip Constructs in Microtitre Plates for Drug Discovery

N.P. Macdonald, A. Menachery, J. Reboud and J.M. Cooper

Supplementary Information

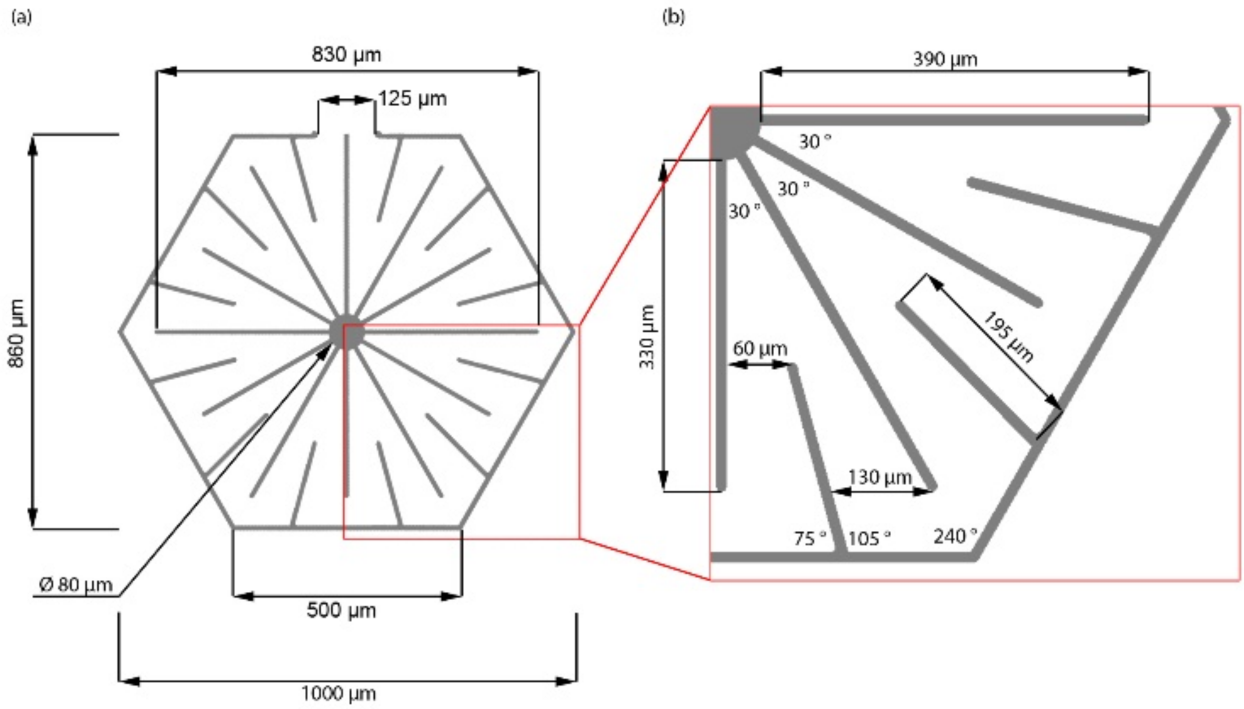

Figure S1. Electrode design

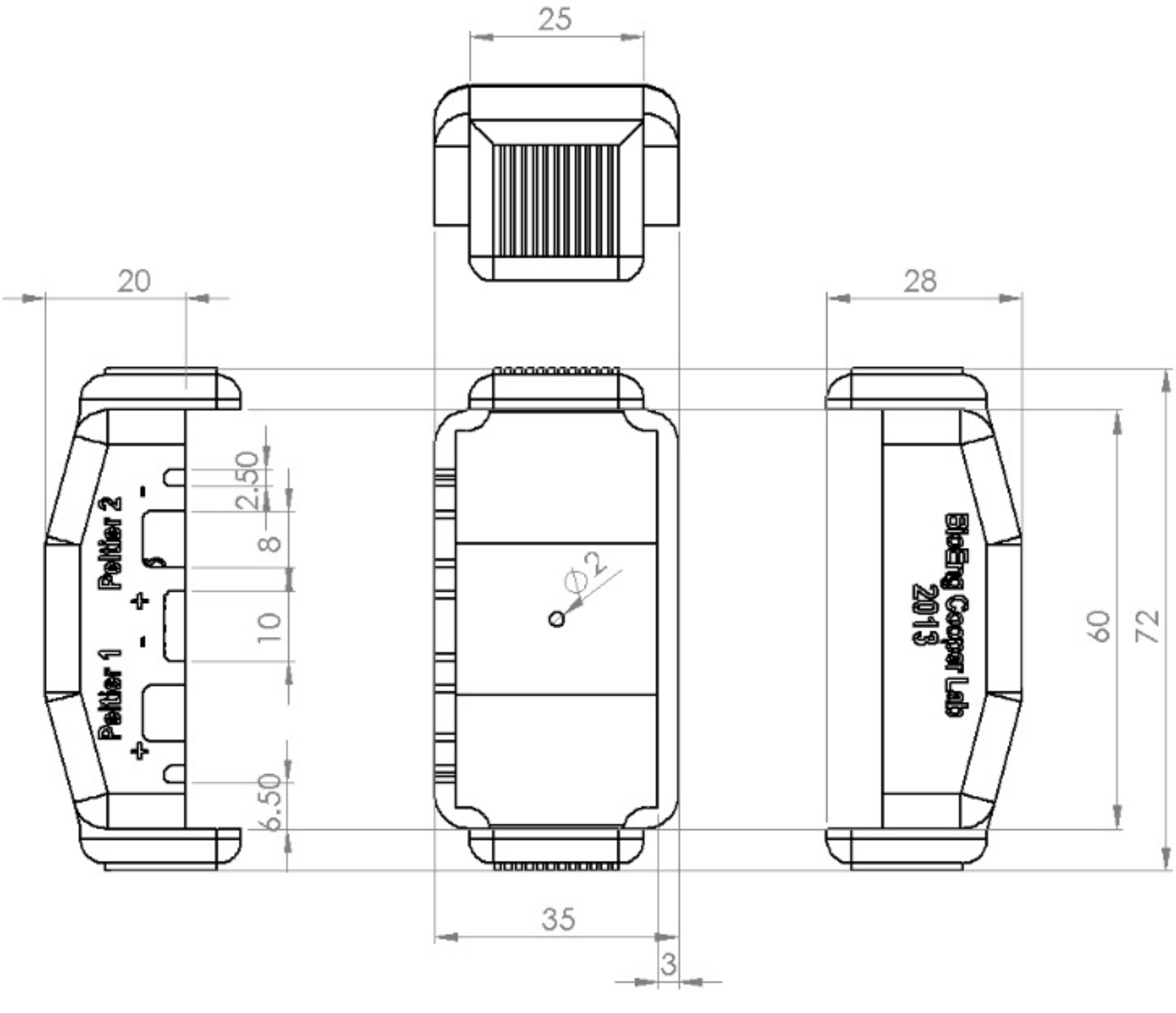

Figure S2. 3D printed cover design

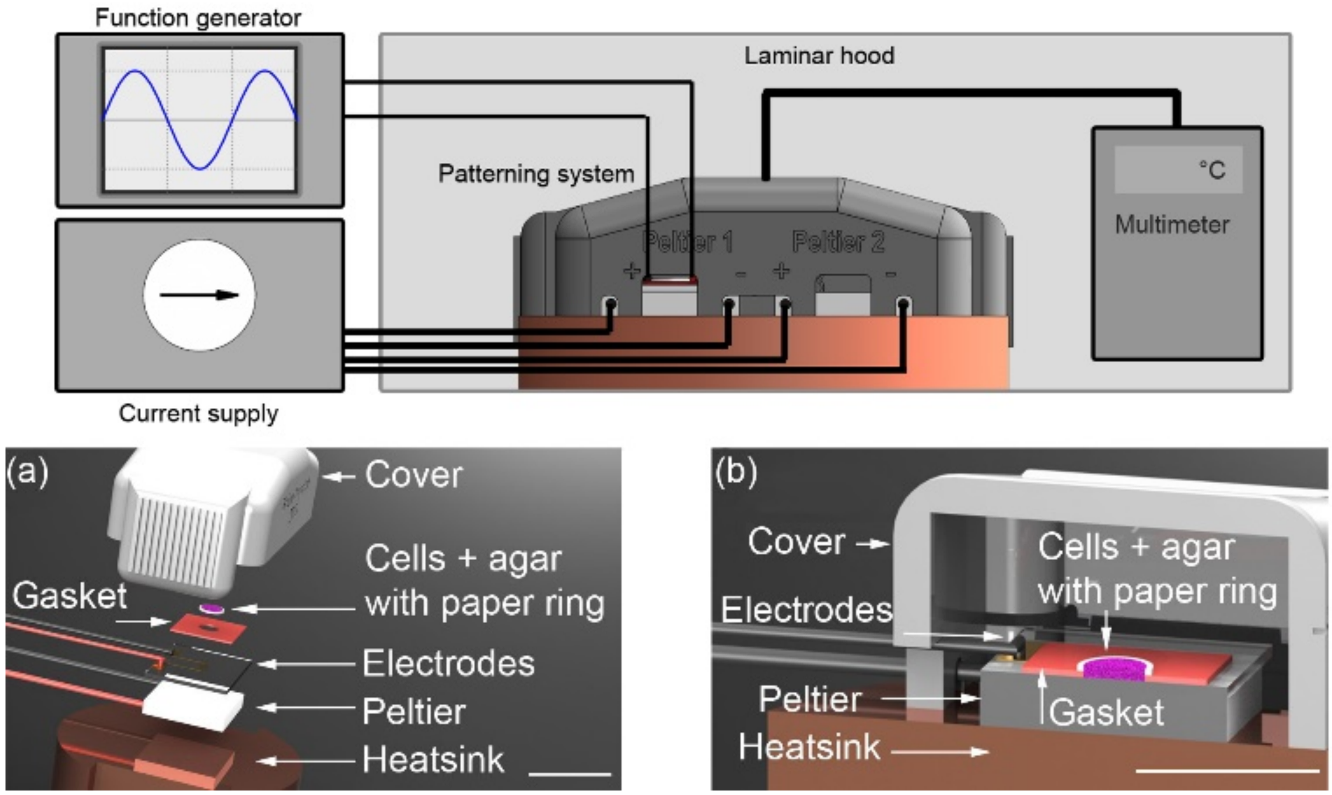

Figure S3 – systems view.
